# Supplementary material for: Isoscapes of remnant and restored Hawaiian montane forests reveal differences in biological nitrogen fixation and carbon inputs
Source: PeerJ. 2023 Jun 6;11:e15468. doi: 10.7717/peerj.15468 (PMC10252893; doi:10.7717/peerj.15468)
Supplement: Supplemental Information 1 [file peerj-11-15468-s001.pdf]

Isoscapes of remnant and restored Hawaiian montane forests reveal differences in biological nitrogen fixation and carbon inputs

Christopher B. Wall, Sean O. I. Swift, Carla M. D'Antonio, Gerhard Gebauer, Nicole A. Hynson

*corresponding author: cbwall@ucsd.edu

Supporting Information

Supplemental figures

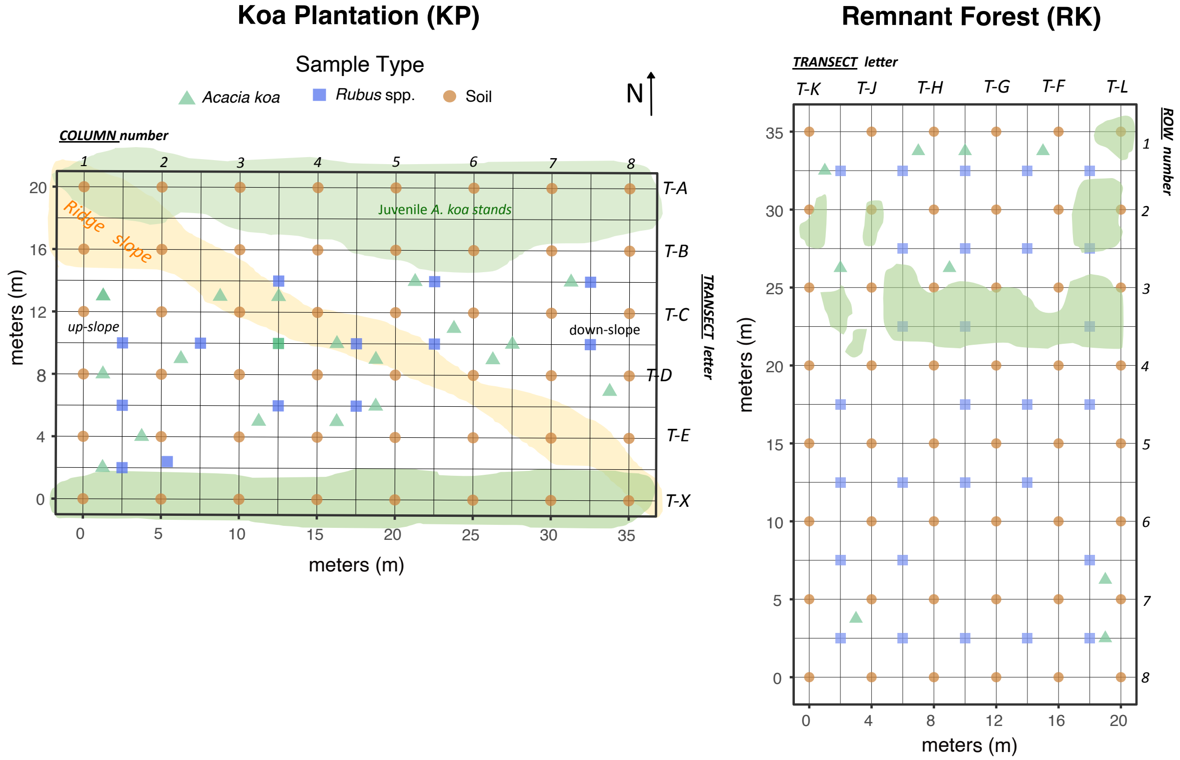

Figure S1. Forest plots (20 × 35 m) in the koa plantation (KP) and the remnant forest (RK) showing locations of sample collection (*Acacia koa* and *Rubus* spp. leaves, soil samples), slopes within the Koa Plantation, and patches of juvenile *A. koa* where samples were not collected.

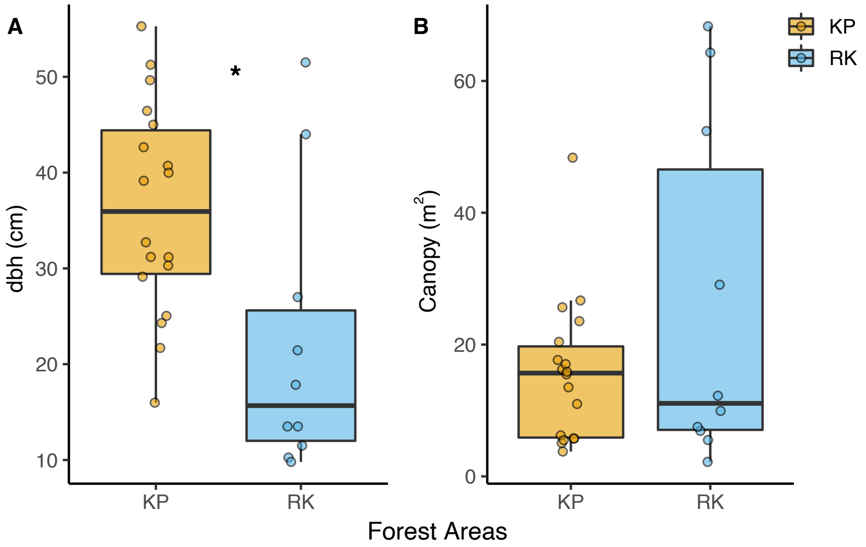

Figure S2. (**A**) Box plots of *Acacia koa* tree diameter at breast height (dbh), and (**B**) *Acacia koa* tree canopy area (m^2) in the koa plantation (KP) and remnant (RK) Hakalau forest areas (*n* = 18 [KP] and 10 [RK]). Asterisks indicate significant plot effects ($p<0.05$).

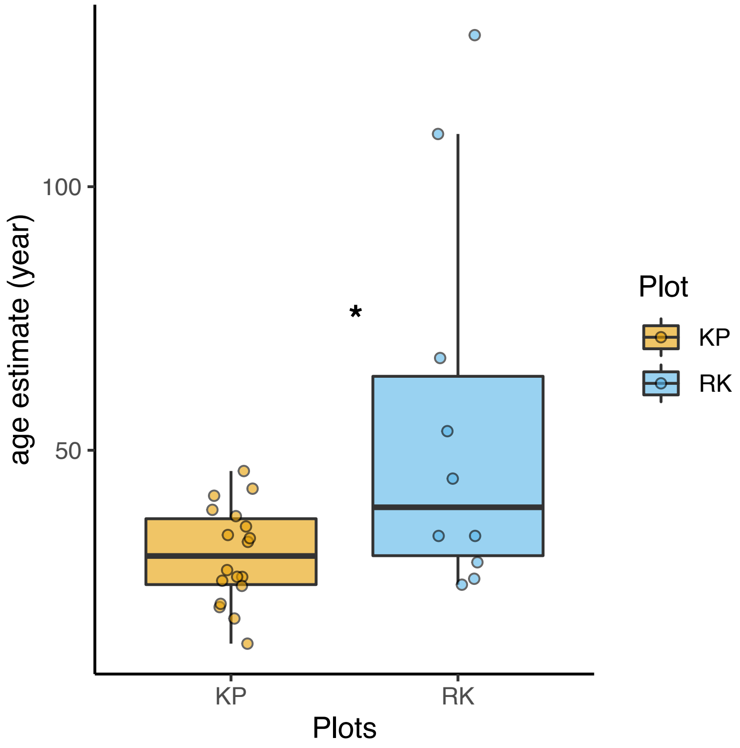

Figure S3. Box plot estimates of *Acacia koa* age in the koa plantation (KP) and remnant forest (RK) using diameter at breast height-based growth rates of 12 mm (KP) and 4 mm per year (RK). Asterisks indicate significant plot effects (p<0.05).

Table S1. Nonparametric tests of forest type (koa plantation vs. remnant forest) effects on *Acacia koa* diameter at breast height, *A. koa* forest canopy area, and *A. koa* age estimates. Forest type effects on the distribution of interpolated values in nitrogen and carbon isoscapes generated from soil or foliar samples.

Variable	*Effect*	*U*	*Z*	*Effect-size r*	*p-value*
Diameter at breast height (dbh, cm)	Forest type	315	2.589	0.489	**0.010**
Canopy area (m^2)	Forest type	252	-0.432	0.082	0.666
dbh-estimated age (years)	Forest type	220	-1.966	0.372	**0.049**
Isoscape: soil					
$\delta^{15}N$	Forest type	4.743×10^8	-66.868	0.281	**<0.001**
$\delta^{13}C$	Forest type	8.194×10^8	116.006	0.487	**<0.001**
Isoscape: foliar					
$\delta^{15}N$	Forest type	1.429×10^8	-129.769	0.637	**<0.001**

U = U-statistic; *Z* = Z-value. Significant effects (p<0.05) are in bold.

Table S2. Linear models testing the effects of forest (remnant forest vs. koa plantation) and sample types (*Acacia koa* leaves, *Rubus* spp. leaves, soil) on response variables.

Variable	*Effect*	*SS*	*df*	*F*	*p-value*
$\delta^{15}N$	Sample type	670.48	2	336.862	**<0.001**
	Forest type	14.07	1	14.138	**<0.001**
	Sample × Forest	3.64	2	1.828	0.164
	Residuals	159.23	160		
$\delta^{13}C$	Sample type	1075.09	2	635.030	**<0.001**
	Forest type	56.56	1	66.823	**<0.001**
	Sample × Forest	2.24	2	1.322	0.269
	Residuals	135.44	160		
Total nitrogen (mmol/gdw)	Sample type	17.544	2	139.520	**<0.001**
	Forest type	0.165	1	2.621	0.107
	Sample × Forest	0.188	2	1.492	0.228
	Residuals	10.060	160		
Total carbon (mmol/gdw)	Sample type	16428.6	2	763.903	**<0.001**
	Forest type	98.6	1	9.166	**0.003**
	Sample × Forest	17.2	2	0.801	0.451
	Residuals	1720.5	160		
C:N	Sample type	929.10	2	107.344	**<0.001**
	Forest type	1.06	1	0.245	0.621
	Sample × Forest	8.84	2	1.022	0.362
	Residuals	692.43	160		

SS = sum of squares; *df* = degrees of freedom. Significant effects ($p<0.05$) are in bold.

Table S3. Model parameters used to perform automatic kriging and generation of variograms for spatial interpolation in the koa plantation (KP) and remnant forest (RK).

isoscape	*metric*	*site*	*model*	*nugget*	*sill*	*range (m)*	*sserr*	*kappa*
soil	$\delta^{15}N$	KP	Matern	0.00	1.44	3.43	0.01	10.00
soil	$\delta^{15}N$	RK	exponential	0.00	1.00	3.00	0.14	–
soil	$\delta^{13}C$	KP	Matern	0.24	1.17	5.09	0.35	10.00
soil	$\delta^{13}C$	RK	exponential	0.00	0.80	3.00	0.12	–
foliar	$\delta^{15}N$	KP	exponential	0.20	0.40	3.00	0.12	–
foliar	$\delta^{15}N$	RK	exponential	0.01	0.82	5.00	0.17	–

isoscape = input sample type used in the spatial interpolation; *metric* = input data type used in isoscapes; *model* = best fit model iteratively selected using R package automap (Hiemstra et al. 2009); *nugget* = minimum semivariance; *sill* = maximum semivariance; *range* = maximum distance, in meters for the current study; *sserr* = sum of squares between sample and fitted variogram; *kappa* = smoothing parameter in Matern (Ste) model.
